# Supplementary material for: Patterns of Intron Gain and Loss in Fungi
Source: PLoS Biol. 2004 Nov 30;2(12):e422. doi: 10.1371/journal.pbio.0020422 (PMC532390; doi:10.1371/journal.pbio.0020422)
Supplement: Table S1 — Also available at http://genes.mit.edu/NielsenEtAl/. (4.3 MB ZIP). [file pbio.0020422.st001.zip › NielsenEtAl/html/897.html]

AN6845.1.NCU08804.1.MG08460.1.FG05096.1


```
 CLUSTAL W (1.82) Multiple Sequence Alignments - Introns Inserted


Sequence 1: NCU08804.1	608 aa
Sequence 2: MG08460.1	477 aa
Sequence 3: AN6845.1	471 aa
Sequence 4: FG05096.1	507 aa
Alignment Length: 611 aa
Number Identitical Residues: 116 aa
Alignment Score (without introns) 7034


MG08460.1 	-----------------------------MSSRDTQPMAIEP---------------AMT
NCU08804.1	MDGWIQSTEPARKITGDDINIELADYLPAQNNVSKSSSVIEPTTEPPLHAESNNTGTANP
FG05096.1 	-----MATDVGDEERAHLLGISPSPSPLPTGVITTPYQPLDQ---------------DES
AN6845.1  	-------------------------------------MESEP------------------
          	                                        :                   

MG08460.1 	AEVIDLDP----PPTRYRDAVPRSLELRLYVSHFLSTWNSRLFEFAAVLFLASVFHDTLL
NCU08804.1	AGDNDLPPSSTSPPVPIPIGISRSLTLRLYTSHFLSTWNSRLFEAGVVYFLASIFPDNLL
FG05096.1 	ANQDDNDS----SSTHSSEWTVSQSARRLYVSHTLSTWNSRVFEFGSVLYLASIFPGTLM
AN6845.1  	-----LVP----HEAPSSSPASNRVLLRLYTSHFLSTWNSRMFEFGAVVFLASIFPGTLL
          	       .      .            ***.** *******:** . * :***:* ..*:

MG08460.1 	PMSVYALARSGAAIVFGQSIGSWIDRGDRLAV~VRVSIV~GQRLAVVVSCGLFWALELRG
NCU08804.1	PVSLYALSRNLAAILFAVPVGHWIDTAHRLTV~VRASIV~GQRLAVAASCGLFWALLELR
FG05096.1 	PLAVYSMIRGAAAIALSSWVGSYIDRNDRLKT0V----V~SQRLVVTVSCAIFLVLIKVR
AN6845.1  	YASVYALVRSLFAVLFSSWLGSLVDRTDRLSA~IRHSII1WQRIPVAVSCVCLGLLLRSP
          	  ::*:: *.  *: :.  :*  :*  .** . :  : :  **: *..**  :  *    

MG08460.1 	HGHWMDSPGLGDGLFALAVVLACVEKLCAVMNLVSVERDW~VVIITEGNELTRR-~----
NCU08804.1	LG-WRQSP-LVDGLFGASVLLACVEKLAAGVNLIAVERDW~VVVITQGNEEARRK~MNAR
FG05096.1 	TM----PHEVRVGLLAVSIIMACIEKLAAIMNFVSVERDW0VIVVARSDTTALRR1----
AN6845.1  	PG------HLSRLLFIALVLLAGVEKLAATVNTVSVERDW0AVVISETLLVSRKD1LNAS
          	         :   *:   :::* :***.* :* ::***** .::::.    : :   .: 

MG08460.1 	-------------------V1AAASTLLAIWTTLAMNILSVAVEYICIAQ0VYKHVPALA
NCU08804.1	MRRIDLFCKLLGPLTV-ALV~AAASVRAAVYATLGMNLASVMVEYLCIET~VFRRVPGLG
FG05096.1 	------------------LV~DGFSTETAILVNLIMNCASVVIEYFTIAR0VYYQVPALQ
AN6845.1  	MRRIDLFCKLVAPVVVSLMD~GLLSTKVAIWAVLGVNVTVVLVEYFAIAG0VYHSVPQLD
          	    .  ..  ..   :       *.  *: . * :*   * :**: *   *:  ** * 

MG08460.1 	VQREAGNAPAHS-------------GAPASTRSAAALS-WVKSMAYR-VLPIESFPFYFH
NCU08804.1	RPAPPGKSVGQSLETLSGAVLRRDGGAATDGRTPFQLGNWIRSVSWRKLLMIPSLRLYFG
FG05096.1 	YPKNAPRTTALD---------------DGNRQNPLASVKLVLKKTFD------DLRLYFT
AN6845.1  	RSHERQAADDEE---------------DEQQPLEEHLAQNIFAQYIR--RTVAPWREYVG
          	       :   .                 .        . :                *. 

MG08460.1 	HAAFLPSFSLALLYLTVLSFSGQMITYLVSVGYSSLHVGVARTVSTVFELSATWIAPRLM
NCU08804.1	HPAVIPSLALSLLYLTVLSFSGQMLTYLLASNLNLWQVGIIRGISTIFELSATWIAPRLM
FG05096.1 	HPVFFPSFAVALLYCTVLSFGGVMVTYLLSSGYNSIQIAAMRTISVAFEVLATWIGPWLM
AN6845.1  	QSVFLASFALSLLYLTVLSFGPTMVTFLLHSGFSSLDVSAMRIGAVLTEISGTWMAPLLM
          	:...:.*::::*** *****.  *:*:*:  . .  .:.  *  :.  *: .**:.* **

MG08460.1 	KRIGIVRGGIWSLCWQMAWLAAGVAWFFADAS-GKGTNSIASATGIVVAVALSRIGLWGY
NCU08804.1	KRIGVLRTGLWSITWQVTWLAGGVSCFFYYYGKGYEATSLMTAVGLVVAVAFSRVGLWGF
FG05096.1 	KKIGPVRAGLWFLSWQLGCLSIGISIFWRYAD-----NVLVSTLALVGSSMFSRVGLWGV
AN6845.1  	DRIGPIRSGLWFLNWQFGTLAAAVAAFAFASD-----RPRLVAGCLITGVALSRLGLWGF
          	.:** :* *:* : **.  *: .:: *    .          :  :: .  :**:**** 

MG08460.1 	DLCAQNIIQD0EVESEYRGTFSTVEASFQNLFELLSYASTIVFSRPDQFQWPIVISVVAV
NCU08804.1	DLSVQNIVQD0EVEDNRRGIFSSVEASFQNMFDMLAWALTIIWPNPNSFQWPIVISVAAV
FG05096.1 	DLSAQVIIQE0EVEAQNRGAFSAVEASWQHVFEMYAYTSTIIFSSPSEFHNPTALSVAAV
AN6845.1  	DLSVQFLVQE0NIDPSSRARFSATEMALQSVFEMISFATTIFFADPDQFKYPVYISYGAI
          	**..* ::*: ::: . *. **:.* : * :*:: ::: **.:. *..*: *  :*  *:

MG08460.1 	YTAGGLYTAFVRRRRGHLFHAPPCMGDKRG~TL---------------------------
NCU08804.1	YAAGGLFAHFLRRRRGHLLHPPACIKAKHL1CQYGRRPQGHDRNVRISCTWQANQALTAL
FG05096.1 	LFAWVLYSLFVKKRRGHLVHWPTCLSAEKQ~QAAAIDGVLESMHSRRREGPGGVA-----
AN6845.1  	ALAAVCFAAYVRRERGHLIHTSKCMGDSRL~-----------------------------
          	  *   :: ::::.****.* . *:  .:                               

MG08460.1 	-------------------
NCU08804.1	KCADDSAIQTNVVASRLTS
FG05096.1 	-------------------
AN6845.1  	-------------------
          	
```
